# Precision treatment exploration of breast cancer based on heterogeneity analysis of lncRNAs at the single-cell level

**DOI:** 10.1186/s12885-021-08617-7

**Published:** 2021-08-13

**Authors:** Yan Zhang, Denan Zhang, Qingkang Meng, Ziqi Liu, Hongbo Xie, Lei Liu, Fei Xu, Xiujie Chen

**Affiliations:** 1 College of Bioinformatics Science and Technology, Harbin Medical University, Harbin, 150081 Heilongjiang Province P. R. China; 2 Department of Pharmacy, The First Affiliated Hospital, Harbin Medical University, Harbin, 150001 Heilongjiang Province P. R. China

**Keywords:** Precise treatment, Breast cancer, Heterogeneity analysis, Single cell sequencing, LncRNA

## Abstract

**Background:**

Breast cancer (BC) is a complex disease with high heterogeneity, which often leads to great differences in treatment results. Current common molecular typing method is PAM50, which shows positive results for precision medicine; however, room for improvement still remains because of the different prognoses of subtypes. Therefore, in this article, we used lncRNAs, which are more tissue-specific and developmental stage-specific than other RNAs, as typing markers and combined single-cell expression profiles to retype BC, to provide a new method for BC classification and explore new precise therapeutic strategies based on this method.

**Methods:**

Based on lncRNA expression profiles of 317 single cells from 11 BC patients, SC3 was used to retype BC, and differential expression analysis and enrichment analysis were performed to identify biological characteristics of new subtypes. The results were validated for survival analysis using data from TCGA. Then, the downstream regulatory genes of lncRNA markers of each subtype were searched by expression correlation analysis, and these genes were used as targets to screen therapeutic drugs, thus proposing new precision treatment strategies according to the different subtype compositions of patients.

**Results:**

Seven lncRNA subtypes and their specific biological characteristics are obtained. Then, 57 targets and 210 drugs of 7 subtypes were acquired. New precision medicine strategies were proposed according to the different compositions of patient subtypes.

**Conclusions:**

For patients with different subtype compositions, we propose a strategy to select different drugs for different patients, which means using drugs targeting multi subtype or combinations of drugs targeting a single subtype to simultaneously kill different cancer cells by personalized treatment, thus reducing the possibility of drug resistance and even recurrence.

**Supplementary Information:**

The online version contains supplementary material available at 10.1186/s12885-021-08617-7.

## Introduction

Breast cancer (BC) is one of the most common causes of cancer-related deaths in women [1, 2]. Chemotherapy is one of the chief means to treat BC, but its clinical outcomes vary largely [3]. Studies have shown that the poor prognosis of BC is mainly because of intratumoral heterogeneity [4]. Accurate subtyping of BC can better analyze intratumoral heterogeneity, make tumor diagnosis more accurate, and make the prognostic difference of subtypes more significant. The treatment strategies explored based on precise classification can greatly improve the effect of treatment and reduce the chance of treatment failure and tumor recurrence.

Recently, commonly used subtyping strategies of BC have mostly been based on histological analysis, and multigene signatures provide biological insight and risk stratification in BC [5–8]. Among them, intrinsic molecular subtypes, defined by the mRNA expression of 50 genes (PAM50), including luminal A, luminal B, HER2-enriched, basal-like and normal-like, have been widely recognized and applied [9–11]. Treatment outcomes and prognoses of PAM50 subtypes are different [12–14]. Luminal A subtype has the best prognosis, while the prognosis of TNBC (triple-negative breast cancer) subtype is worst [15–17]. (In clinical research, TNBC is often used to approximately replace the basal-like subtype, as they overlap approximately by 80% [18, 19]). These studies suggest that subtyping methods based on histological multicell levels cannot reveal complete intratumoral heterogeneity. PAM50 subtypes reflect the average status of molecular characteristics of cells in the tissue. Some tumor cells may be resistant to drugs, resulting in cancer progression or even recurrence, which can explain the failure of chemotherapy to some extent.

In recent years, the development of single cell sequencing technology has provided a great opportunity to excavate intratumoral heterogeneity information of BC for precise treatment due to the following advantages: bulk sequencing technologies provide the average level of expression of genes distributed in multiple cells of tissue [20], and information on low-abundance genes and intratumoral heterogeneity will be lost [21, 22]. However, single-cell sequencing technologies can reveal the gene expression status of single cell, detect differences in genetic information between cells, and help to explain the mechanism of cancer progression [23, 24]. Therefore, the subtyping approaches based on single cells are better at revealing intratumoral heterogeneity than subtyping approaches based on multiple cells [7, 25].

However, the current classification of BC at the single-cell level mainly focuses on mRNAs/genes. Woosung Chung et al. used a single-cell gene expression profile to divide 11 BC patients into ER+, HER2, and TNBC subtypes [26], but it was not much different from the PAM50 subtypes. Although many contributions have been made to gain insight into the molecular characteristics of BC cells, room still remains for further study.

Increasing evidences show that lncRNAs play important roles in cell differentiation, migration and apoptosis by regulating gene expression patterns, and lncRNA expression is more tissue-specific, developmental-stage-specific and cell-type-specific than mRNA expression, suggesting that lncRNAs may become key regulators of cell fate and cell-type-specific functions [27–32]. Based on this, we suspect that lncRNAs can also be used for BC subtyping. Subtyping individual cells based on lncRNA expression profiles may be more conducive to revealing intratumoral heterogeneity and obtaining more accurate subtyping results with precise diagnosis and significant prognosis to further explore more effective treatment strategies.

Because subtyping methods of BC based on single-cell lncRNA sequencing data can show the molecular characteristics of single cells, we hypothesize that multiple subgroups/subtypes of cancer cells can be found in the same patient, which may lead to treatment failure or tumor recurrence. We should develop more accurate and effective clinical treatment measures based on the advantages of single-cell analysis. For patients with different subtypes, different drugs should be selected for treatment. These drugs can be targeted multi-subtype drugs or a combination of targeted single-subtype drugs to improve the medication efficiency and reduce the toxicity and side effects.

**Fig. 1:**
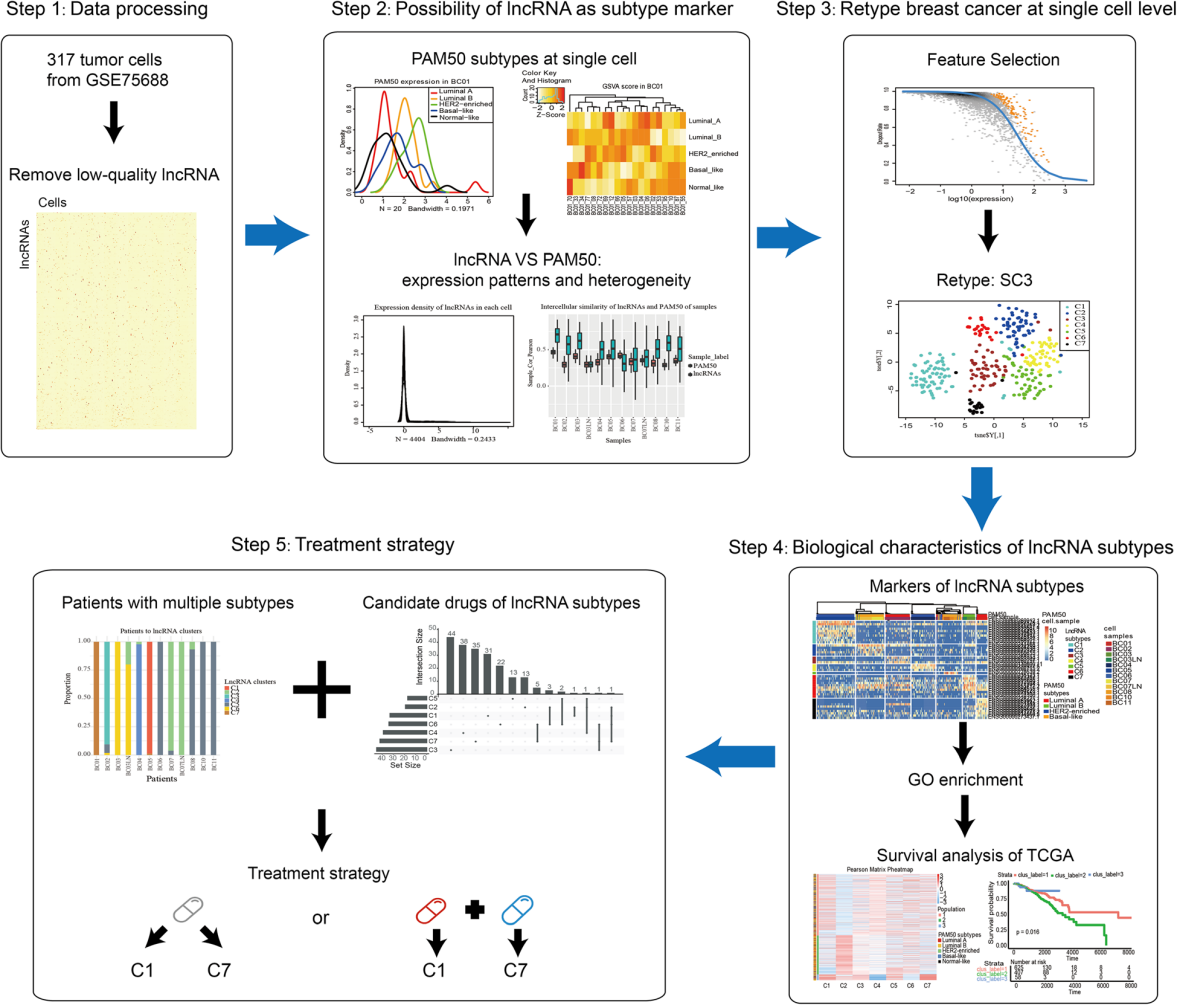
Workflow diagram

In this article, we combined single-cell sequencing data of BC and lncRNA expression profile to subtype 317 tumor cells from 11 BC patients in order to more precisely discover single-cell subtypes and explore precise treatment strategies for patients with multiple subtypes (Fig. 1)

## Methods

### Data processing

Single cell expression profiles (GSE75688) of lncRNAs and PCGs (protein-coding genes) with processed TPM values in 517 cells of 13 samples from 11 BC patients were downloaded from the GEO database [33]. These samples belong to four subtypes of PAM50 subtypes, including luminal A (BC01-BC02), luminal B (BC03), HER2-enriched (BC04-BC06), and basal-like/TNBC (BC07-BC11), while BC03LN and BC07LN collected from regional metastatic lymph nodes belong to luminal B and basal-like, respectively. Tumor cells, stromal cells, and tumor infiltrating immune cells were isolated by the label information of cells downloaded directly from the article of Chung W et.al [26]. The results of cell isolation showed that BC09 cells were all nontumor cells and were not included in the subsequent analysis.

We extracted the expression profiles of lncRNAs and PCGs according to gene type information (lncRNA: 3prime overlapping ncRNA, antisense, lincRNA, processed transcript, sense intronic, sense overlapping; PCGs: protein coding genes) contained in GENCODE [34]. To remove lncRNAs or PCGs with low expression values, the following steps were applied: First, lncRNAs and PCGs with TPM values < 1 in all tumor cells were considered unreliable and removed. Second, lncRNAs and PCGs expressed in < 1% of tumor cells were removed. Then, TPM values were log2-transformed after adding a value of one.

In all, 317 single tumor cells of 12 samples from 10 patients along with 4404 lncRNAs and 15,637 PCGs were applied for the downstream analysis.

### Intratumoral heterogeneity analysis

To illustrate the intratumoral heterogeneity of BC, we compared the similarity of lncRNA expression between cells. The R package Rtsne (version 0.15) [35] was used for the visualization of cell distances in the reduced 2D space of all mixed cells, tumor cells, and nontumor cells to represent the relative positions of the coordinates for comparing the degree of similarity between cells.

### Analysis of PAM50 subtypes at the single-cell level

To show the molecular characteristics of PAM50 subtypes at single cell level, we took the following steps: 1. The R package stats (version 4.0.3) [36] was used to obtain the expression density curves of PAM50 subtypes in 12 samples. The average expression value of signature genes of each PAM50 subtype in each cell was taken as the expression value of this subtype in each cell. 2. The R package GSVA (version 1.38.2) [37] was used to evaluate the expression activity of PAM50 in 12 samples from 10 patients. 3. The R package genefu (version 2.22.1) [38] was applied for PAM50 classification of individual cells.

### Analysis of the expression characteristics of lncRNAs in tumor cells

To explore whether lncRNAs can also be used as markers for subtyping BC, such as PAM50, we compared the expression characteristics of lncRNAs and PAM50 by the following aspects: 1. Expression density of lncRNA/PAM50 in each cell and 2. Expressed cell proportion of each lncRNA/PAM50. And density curve figures were drawn by R packages stats (version 4.0.3).

To further illustrate that lncRNAs are better markers than PAM50, we performed the following steps: 1. The Pearson correlation coefficient between the lncRNAs and PAM50 genes in cells was compared. The larger the Pearson correlation coefficient, the smaller the heterogeneity. 2. Compare the CV (coefficient of variation = standard deviation/mean) of lncRNAs and PAM50 genes, with the CV reflecting the degree of dispersion of expression of lncRNA/PAM50. The larger the CV, the higher the heterogeneity. The Wilcoxon test was used to verify the statistical significance of the above two steps.

### Identification of lncRNA subtypes

To identify subtypes of BC more accurately, and save calculating time and cost, M3Drop [39] was applied to select important lncRNA features that have key effects on subtyping. Since the Michaelis-Menten equation is a nonlinear convex function, the lncRNAs in the upper/right (which means FDR < 0.05) of the Michaelis-Menten model are differentially expressed lncRNAs between cell populations in the data set, which indicates that they have key roles in subtyping. Threshold was set to FDR < 0.05.

Six unsupervised clustering methods, including unsupervised clustering methods developed based on bulk sequencing data, including hierarchical clustering (HC), NMF [40] and unsupervised clustering methods developed specifically for single-cell sequencing data, including SC3 [41], SIMLR [42], RaceID [43] and Seurat [44], were used to perform cluster analysis based on important lncRNA expression profiles of tumor cells.

By comparing the average rouge score (the higher the rouge value, the higher the purity of the cell cluster and the better the clustering method) calculated by the R package ROUGE (version 1.0) [45], we selected the most appropriate clustering method.

### Identification of markers of lncRNA subtypes

SC3 was used to identify the lncRNA markers of each lncRNA subtype, and the threshold was set to auroc≥0.75, *P* < 0.01. In order to verify the representativeness of the obtained lncRNA markers, we performed cluster analysis again by using SC3 based on the lncRNA markers of subtypes, and compared them with the previous clustering results. The indicator adjusted Rand index (ARI) is used to measure the consistency of the two clustering results and is calculated by python module ‘scikit-learn’. The closer the ARI id to 1, the higher the degree of consistency between the two clustering results, and the more representative the identified lncRNA markers are.

### Enrichment analysis

To further clarify the functional characteristics of each lncRNA subtype, we directly identified the differentially expressed genes (DEGs) of lncRNA subtypes and enriched DEGs with GO term functions. The R package scran (version 1.18.5) [46], a method to find candidate marker genes for cell clusters by detecting differential expression between cell clusters, was used to identify DEGs in each subtype, with the screening threshold set as follows: the |logFC| between subtypes was greater than or equal to 1, and the FDR was less than 0.01. Based on these DEGs, the enrichment of GO functions was performed using the R package clusterProfiler (version 3.18.1) [47], and *P* < 0.05 was selected as the threshold of GO terms for significant enrichment.

### Survival analysis

To verify the effect of different subtypes on the survival prognosis of patients, tissue expression profiling data and corresponding patient survival information from TCGA-BRCA was used to perform survival analysis. Since the expression profile of TCGA cannot completely cover 44 lncRNA markers of 7 lncRNA subtypes, we used the DEGs of each lncRNA subtype to calculate the Pearson correlation between TCGA patients and the 7 lncRNA subtypes. Hierarchical clustering was performed based on the correlation matrix to group patients. Kaplan–Meier survival curves of different patient groups were generated using the R package survival (version 3.2.7).

### Candidate drugs for each lncRNA subtypes

To provide novel insight for the clinical treatment of BC based on the characteristics of new lncRNA subtypes and further explore the treatment strategy of BC at the single-cell level, we identified candidate targets and drugs for each lncRNA subtype according to the following steps: after removing the duplicate GO terms between the lncRNA subtypes, the specific GO terms of each lncRNA subtype were obtained. Then, the Pearson correlation between lncRNA markers and genes of specific GO terms was calculated by the R package psych (version 2.0.12), and we selected genes with |cor| > 0.2 [48] and *P* < 0.05 as candidate regulatory targets for lncRNAs. The R package scran (version 1.18.5) [46] was used to identify DEGs between tumor cells and nontumor cells. Intersection of candidate regulatory target genes of lncRNA and DEGs between tumor cells and nontumor cells were served as candidate targets for each lncRNA subtype. Then we downloaded the drug-target information from three databases (DrugBank [49], TTD [50], and Drug Repurposing Hub [51]), integrated candidate targets of each subtype and drug-target interaction information, and selected drugs appearing in more than two databases as candidate drugs for each subtype.

## Results

### lncRNAs as better subtyping markers than PAM50

**Fig. 2:**
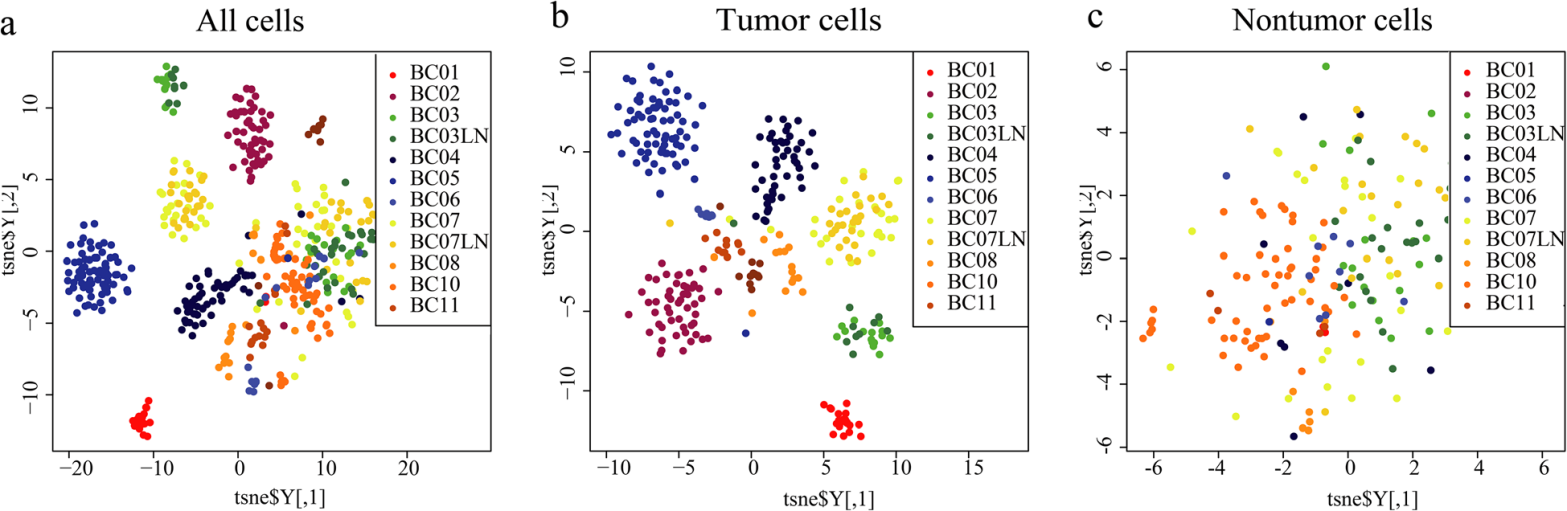
Tsne display of all cells (**a**), tumor cells (**b**), and nontumor cells (**c**) in 11 patients. BC01-BC02: Luminal A, BC03: Luminal B, BC04-BC06: HER2-enriched, BC07-BC08, BC10-BC11: Basal-like/TNBC

Figure 2a, b, and c show that tumor heterogeneity mainly comes from the heterogeneity of tumor cells and Fig. 2b and c show that the heterogeneity of tumor cells is greater than that of nontumor cells. Figure 2b indicate that there is a wide range of heterogeneity between tumor cells even within tumor cells of the same PAM50 subtype. In Fig. 2b, we also found that samples from the same PAM50 subtype were not gathered together at the single cell level. For example, BC01 and BC02 are two separate parts, but both belonging to luminal A, which indicates that there is a wide range of intratumoral heterogeneity in PAM50 subtypes. This shows obvious patient variability, which presents heterogeneity at the patient level.

Based on the above phenomenon, we further evaluated the molecular characteristics of PAM50 subtypes at the single-cell level (Figure S1) by expression density maps of PAM50 subtypes in 10 patients. For all samples except BC06, the corresponding subtype curve cannot be separated from the other three. In particular, BC03, which belongs to luminal B, was highly expressed in the expression density map, and its signature genes wereHER2-enriched. A previous study showed that luminal B subtypes can express ERBB2/HER2 genes [52]. This shows that the PAM50 subtypes do not completely express the heterogeneity characteristics of single tumor cell.

In addition, the expression activity (Figure S2) was displayed to evaluate the expression status of PAM50 subtypes in 10 patients. Taking BC01 as an example, the signature genes of luminal A have different expression trends in different cells (some are highly expressed and some are expressed at low levels), and they cannot be well distinguished from at gene expression of the other three PAM50 subtypes. Moreover, the expression pattern of PAM50 subtypes varies from cell to cell, and each sample has expression characteristics of multiple PAM50 subtypes.

**Fig. 3:**
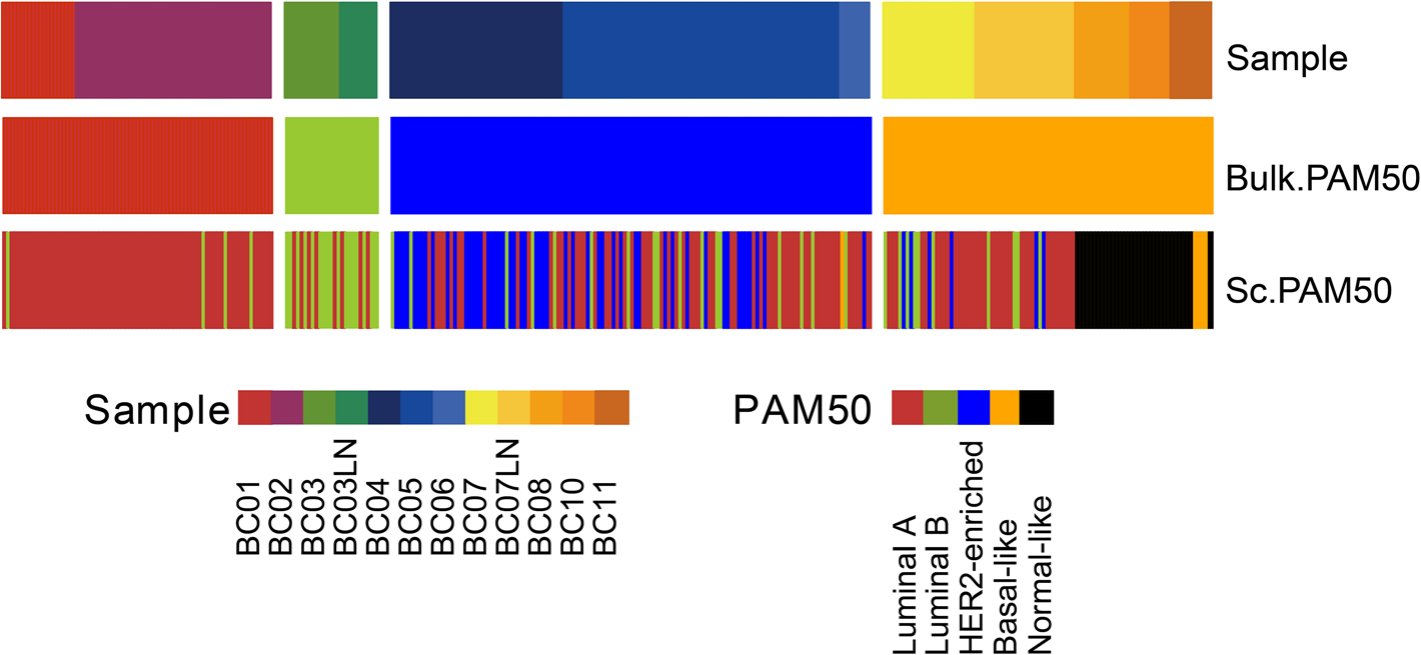
Sample source, bulk PAM50 subtype, predicted PAM50 subtypes of individual cells

Therefore, we further predicted the PAM50 subtype of individual cells. From the PAM50 classification results of individual cells (Fig. 3), multiple cells belonging to one PAM50 subtype at the tissue level will belong to different PAM50 subtypes, which indicates that the effect of PAM50 subtypes is not ideal and cannot reveal the complete intratumor heterogeneity of BC. Therefore, a new subtyping method is needed to analyze the heterogeneity of BC cells.

**Fig. 4:**
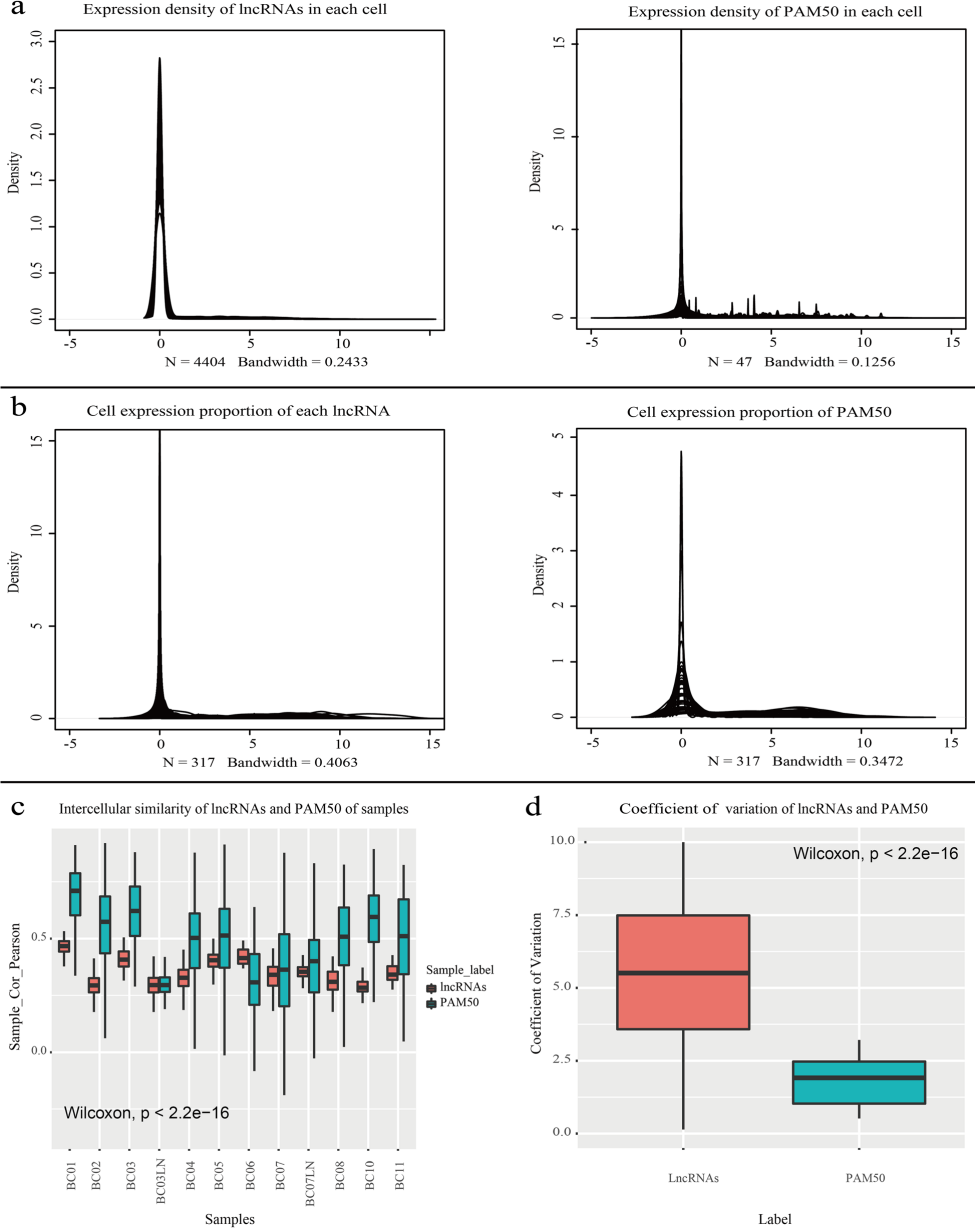
Comparison of the expression consistency of lncRNAs and PAM50. **a** Proportion density distribution of lncRNAs and PAM50 in each cell. The abscissa indicates the level of expression, and the ordinate indicates the density of expressed lncRNAs or APM50. **b** The expression density distribution of each lncRNA and each signature gene of PAM50 in cells. The abscissa indicates the expression level, and the ordinate indicates the density of the expressed cells. **c**. Comparison of expression heterogeneity of lncRNAs and PAM50 in 10 patients. The abscissa represents the samples and the ordinate represents the Pearson similarity between cells. Red represents lncRNAs and green represents PAM50. **d** CV of lncRNAs and PAM50 for 10 patients. The abscissa indicates lncRNAs and PAM50, and the ordinate indicates the CV

We supposed that subtyping markers should meet the following criteria: 1. Markers should be highly expressed in a small number of cells, and expressed close to 0 in most cells and 2. In a single cell, only a small number of markers are highly expressed, and the expression of others is close to 0. Here, we compared the expression consistency of lncRNAs and PAM50 (Fig. 4a-b, Additional file 1) to illustrate that lncRNAs can be similar to PAM50 in characterizing BC cells. Then, we compared the expression heterogeneity of lncRNAs and PAM50 (Fig. 4c-d) to assess whether lncRNAs are more suitable than PAM50 as new markers for subtyping of BC.

From Fig. 4a-b, we found that for each lncRNA expressed in high abundance in a few of cells, PAM50 was too, which indicates that lncRNA is similar to PAM50, that is the expression of lncRNAs in cancer cells is also heterogeneous, and thus, lncRNAs can be used as markers for reclassifying BC cells. In Fig. 4c, except BC03LN and BC06 (their cell number is too small to accurately reflect the overall level of heterogeneity distribution), the intercellular similarity of lncRNAs in other samples is lower than that of PAM50. Figure 4d shows that the CV of lncRNAs is significantly higher than that of PAM50, indicating that the degree of dispersion of lncRNAs between tumor cells is stronger and that the intercellular heterogeneity of lncRNAs is higher than that of PAM50. Therefore, lncRNAs can more significantly represent the heterogeneity of tumor cells, which means lncRNAs might be more suitable as subtyping markers for BC.

### Identification of new subtypes of breast cancer

**Fig. 5:**
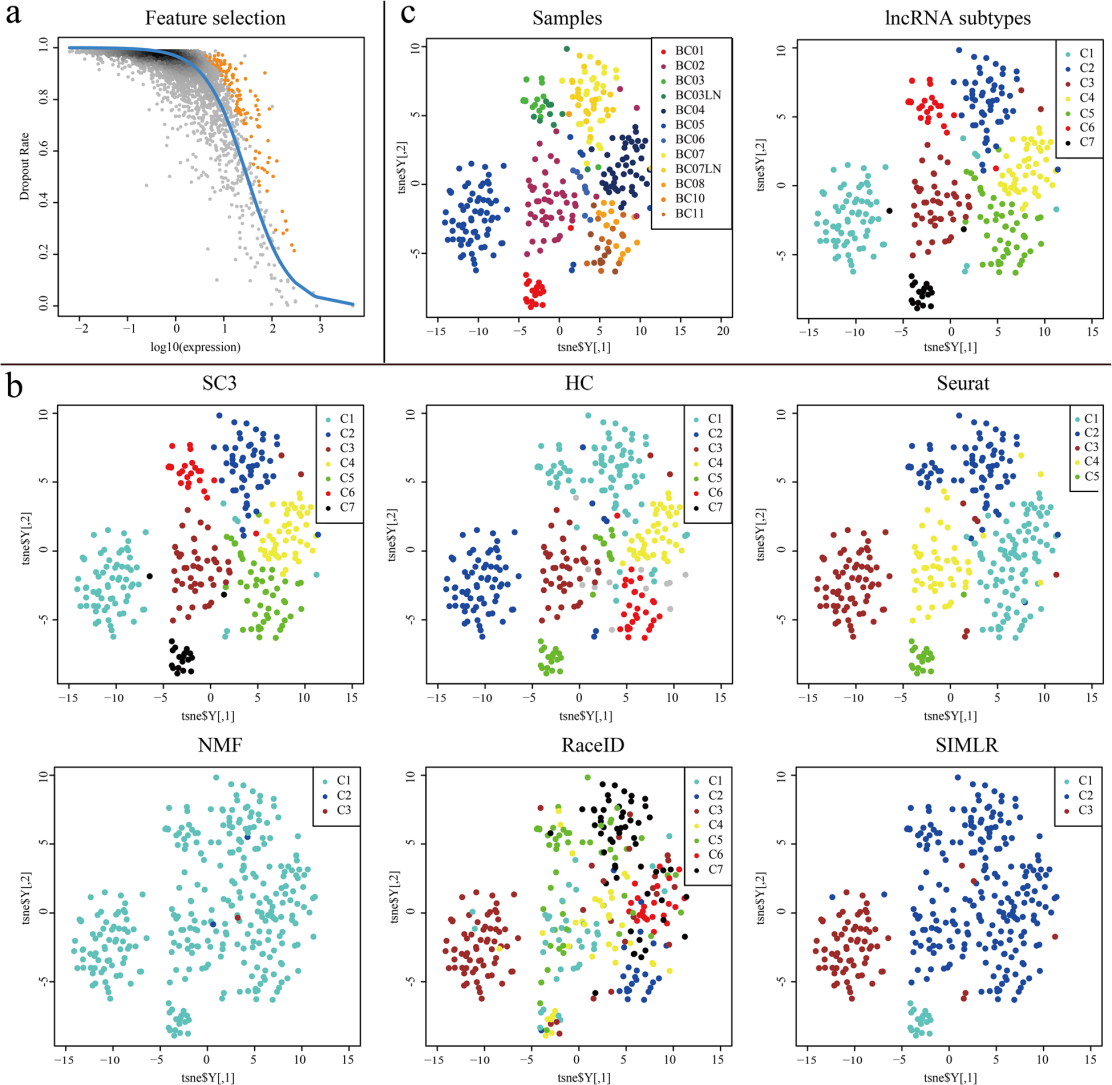
Feature selection and comparison of results of 6 clustering methods. **a** Important lncRNAs selected by M3Drop. The abscissa is the expression value, the ordinate is the dropout rate, the blue line is the fitted curve, and the orange dots represent the important lncRNAs. **b** Tsne dimensionality reduction display and clustering result displays of HC, SC3, SIMLR, NMF, RaceID and Seurat after feature selection. **c** Tsne dimension reduction visualization of PAM50 subtypes and lncRNA subtypes of SC3. BC01-BC02: Luminal A, BC03: Luminal B, BC04-BC06: HER2-enriched, BC07-BC08, BC10-BC11: Basal-like/TNBC

In the expression profile of lncRNAs, some lncRNAs have weak correlation with BC heterogeneity, which will interfere with subtyping results. Therefore, we first identified important lncRNAs. A total of 119 lncRNAs with a multiple correction FDR < 0.05 were obtained (Fig. 5a) for further analysis. Then, 6 clustering methods were performed on important lncRNAs in 317 tumor cells (Fig. 5b).

We prefer to select the method meeting the following conditions: 1. Complete separation between clusters, 2. The number of clusters was at least greater than the number of PAM50 subtypes, and 3. The average rouge score is highest. The average rouge scores of the six methods are 0.999921 (SC3), 0.999641 (HC), 0.994104 (NMF), 0.999166 (RaceID), 0.997553 (Seurat) and 0.998465 (SIMLR), respectively. It can be seen that SC3 is the best. From Fig. 5b, the clustering visualization of SC3 (Figure S3) is most obvious, and the number of clusters (lncRNA subtypes) is 7. In Fig. 5c, it can be seen that the cells of the same PAM50 subtype are not always similar. Tumor cells of the same PAM50 subtype were dispersed in at least two lncRNA subtypes. In the lncRNA subtypes of SC3, except for a few cells, the clustering results of other cells can ensure that the closely related clusters are separated from each other.

**Fig. 6:**
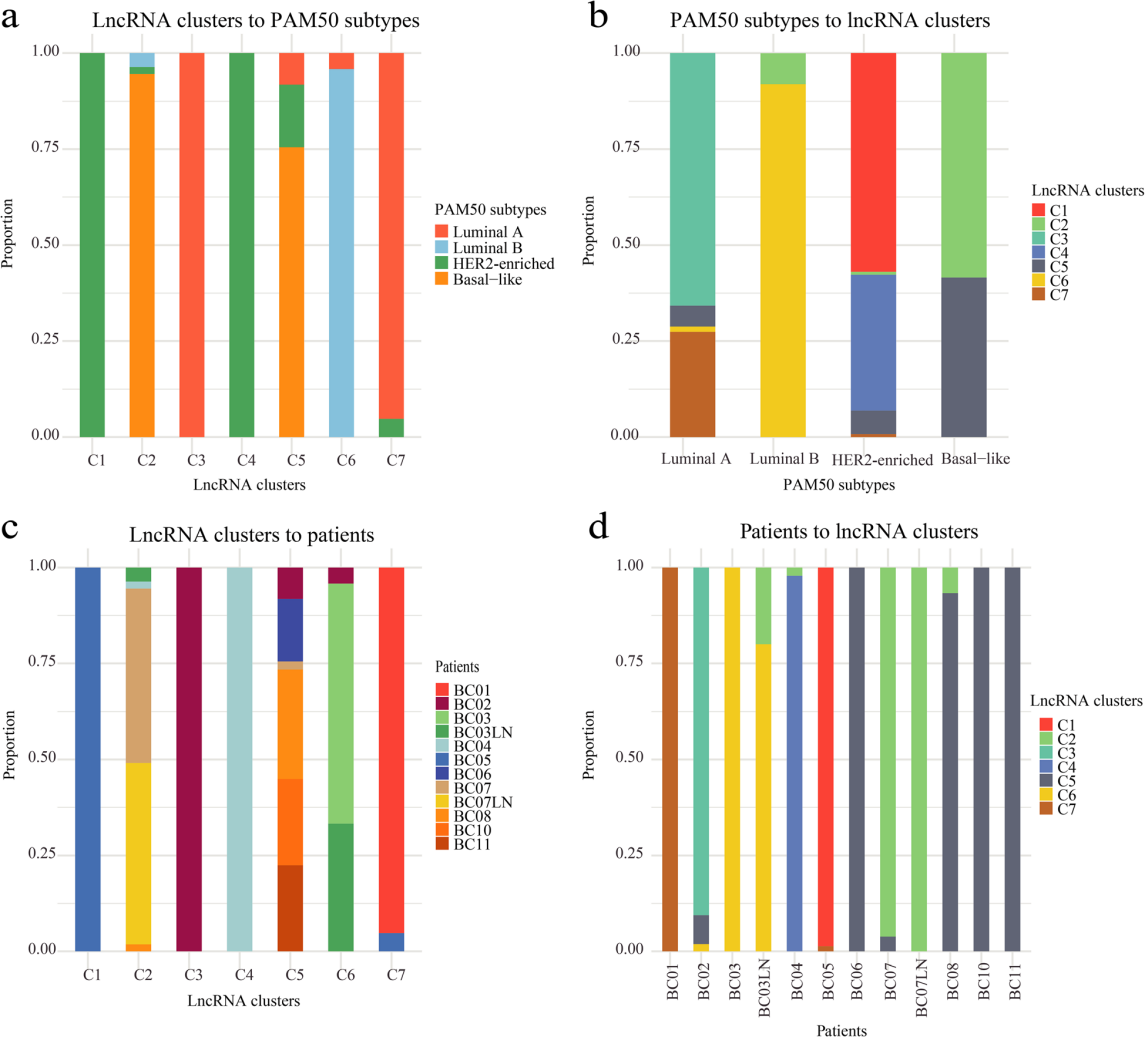
Types and proportions of lncRNA subtypes of patients. **a** Enrichment of lncRNA subtypes in each PAM50 subtype, **b** enrichment of PAM50 subtypes in each lncRNA cluster, **c** enrichment of lncRNA subtypes in each patient, and **d** enrichment of patients in each lncRNA subtype

Resubtyping strategies based on the expression of important lncRNAs better solves the problem of unclear boundaries of subtypes due to intratumoral heterogeneity in BC. Since the gold standard for the molecular classification of BC is PAM50 subtypes, we compared lncRNA subtypes with PAM50 subtypes (Fig. 6a-b). Compared with PAM50 subtypes, lncRNA subtypes can reveal the phenomenon of multiple lncRNA subtypes in each PAM50 subtype at the single-cell level. We have counted the types and proportions of lncRNA subtypes in each patient (Fig. 6c-d). Some patients contain cells of multiple lncRNA subtypes at the same time, which reflects the high resolution of typing based on the single cell sequencing data. For example, patients BC02, BC03, BC04, BC5, BC07 and BC08, have at least two PAM50 subtypes. For these patients, the cell number of each lncRNA subtype was different. We defined the lncRNA subtype with a highest proportion of cancer cells as the major/dominant subtype, and other lncRNA subtypes were minor subtypes. Detailed information about the subtypes of each patient is shown in Additional file 2.

### The lncRNA markers of new lncRNA subtypes

**Fig. 7:**
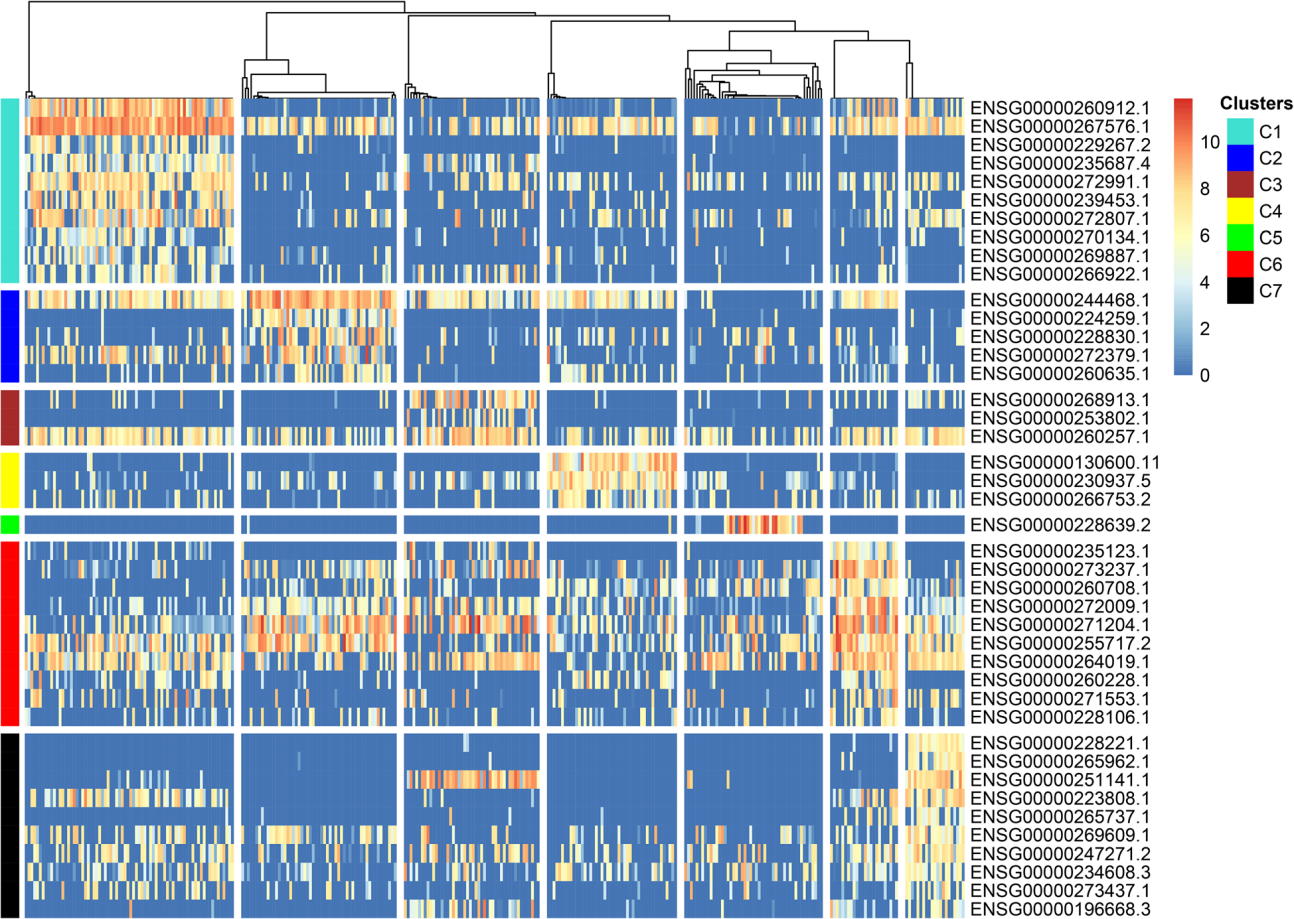
Heatmap of lncRNA marker expressions of lncRNA subtypes

Forty-four lncRNA markers, including 10, 5, 3, 3, 1, 10, and 12 lncRNAs of 7 lncRNA subtypes, were obtained (Fig. 7, Additional file 3). SC3 was reused to perform unsupervised clustering for 317 tumor cells based on 44 lncRNA markers to assess the representativeness of lncRNA markers by comparing the number of clusters with those of the previous lncRNA subtypes. The optimal number of clusters of markers is the same as the previous number of clusters. The consistency of the marker clustering results with the previous lncRNA subtyping results was high (ARI = 0.928). This illustrates that the identified lncRNA markers of each subtype are sufficient to distinguish the new lncRNA subtypes.

### Biological characteristics of lncRNA subtypes

To better characterize the biological functions of each lncRNA subtype, we performed enrichment analysis of DEGs of each lncRNA subtype to obtain their specific functional characteristics. When the threshold was |logFC| ≥ 1 and FDR < 0.01, the numbers of DEGs of each subtype identified were 427, 332, 652, 419, 248, 526 and 942 (Additional file 4). Significantly enriched GO terms were selected at *P* < 0.05 and are shown in Additional file 5. Each subtype has specific functional characteristics. For example, C3 is related to transcriptional functions, including RNA degradation, RNA splicing, RNA stability regulation, and RNA localization [53]. The RNA-binding protein NONO that promotes BC proliferation [43] is involved in nuclear speck of CC (cellular component) and RNA splicing of BP (biological process).

### Survival validation based on TCGA patients

**Fig. 8:**
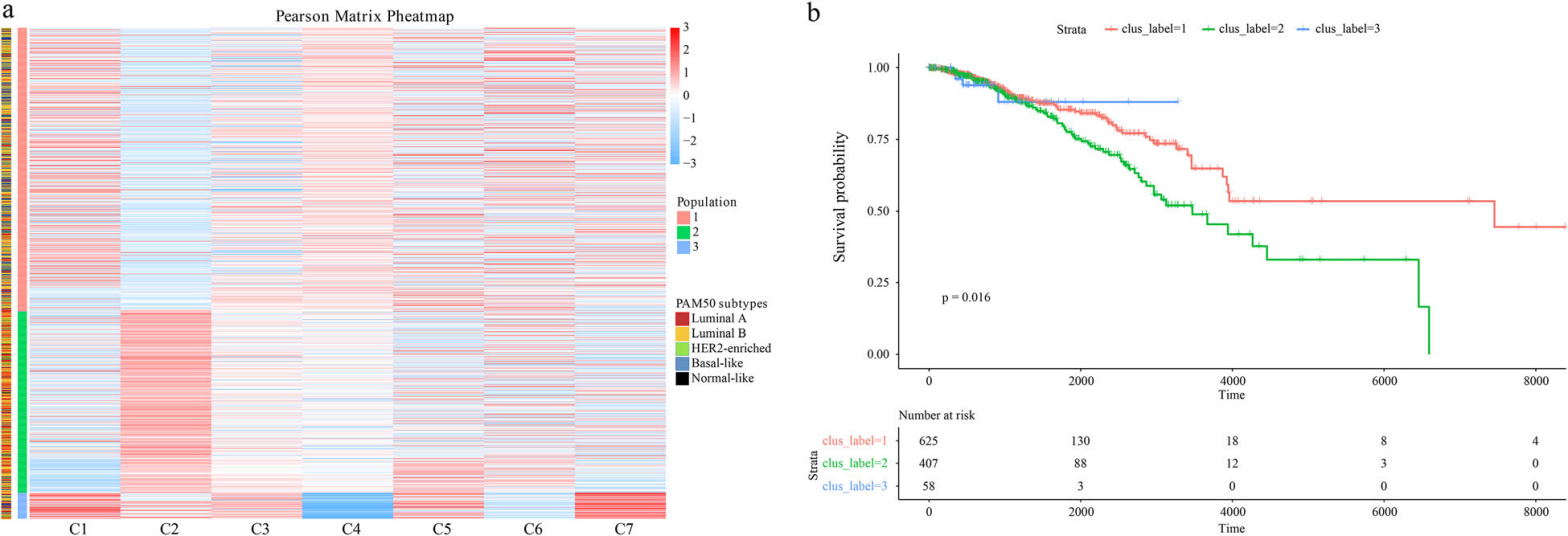
Results of survival analysis. **a** Heatmap of the Pearson coefficient between TCGA patients and 7 lncRNA subtypes. **b** The survival curves of 3 populations of TCGA patients

Since there is no survival time information in our data, we downloaded a new set of data from 1092 BC patients from TCGA for survival analysis. The Pearson correlation between the patients and the 7 lncRNA subtypes is shown in Fig. 8a, and patients were divided into 3 populations. Population 1 has the highest correlation with C4, medium correlation with C1 and C3, and the lowest correlation with C2, and the other ncRNA subtypes have no difference from population 2. In contrast, population 2 has the highest correlation with C2 and the lowest correlation with C1 and C3. Population 3 has the highest correlation with C1 and C7, and the lowest correlation with C4 and C6. The survival curves of the 3 populations are shown in Fig. 8b. Population 2 had the lowest survival rate and population 3 had the highest survival rate.

To verify the difference in survival curves among the 3 populations, we tested the impact of lncRNA markers of 7 lncRNA subtypes on the survival or prognosis of patients or the development of BC through the literatures.

For population 2, there is no literature to show the correlation between lncRNA markers of C2/C3 and BC, and thus, so we focused on the impact of lncRNA markers of C1 on BC. LINC00993, a lncRNA marker of C1, acts as a tumor suppressor [54]. The lowest correlation with C1 confirms that population 2 has the lowest survival rate.

For population 3, we focused on markers of C1, C4 and C6 because there are no reports about the relationship of markers of C7 and BC. H19 is a lncRNA marker of C4. Studies show that H19 overexpression in 73% of BC tissues enhances BC cell migration [55]. It mediates the resistance of BC cells to paclitaxel, trastuzumab and tamoxifen for [55–57]. It is associated with poor prognosis in BC patients, particularly in TNBC subtype [58]. DSCAM-AS1 and SNHG1 are lncRNA markers of C6. DSCAM-AS1 promoted the proliferation and invasion of BC cells by reducing miR-204-5p and inhibiting miR-204-5p expression [59]. SNHG1 promotes BC [60] and impedes the immune escape of BC [61]. The highest correlation with C1 and the lowest correlation with C4 and C6 confirm that population 3 has the highest survival rate. However, since the longest survival time of population 3 patients is only 3283 days, the trend of the survival curve in the second half of population 3 cannot be predicted. For population 1, the highest correlation with C4 and medium correlation with C1 verified the medium survival rate of population 1.

### Precision treatment for patients with multiple subtypes

To explore treatment strategies based on the new subtyping result, it is also necessary to identify candidate therapeutic drugs for single-cell lncRNA subtypes. Drugs can indirectly target lncRNAs to exert antitumor efficacy by regulating lncRNA-related pathways or regulators. Therefore, we indirectly targeted lncRNA by identifying drugs that target lncRNA regulatory genes. For 7 lncRNA subtypes, 210 candidate drugs were identified and are shown in Table 1 and Additional file 6. We conducted direct (drug-disease relationships) or indirect (drug-target, target-disease relationships) literature verification on candidate drugs. In addition, the total literature verification rate is 86.2%. We found that some drugs are for only one lncRNA subtype, such as imatinib, which is only for subtype C1, and some drugs are suitable for multiple subtypes, such as canfosfamide, which is a therapeutic drug for the C4, C5 and C6 subtypes. Among these predicted drugs, we found that some drugs are currently used to treat PAM50 subtypes. For example, candidate drugs for C1, including trastuzumab, pertuzumb, lapatinib and sorafenib are commonly used for HER2-enriched subtypes. Fulvestrant, a commonly used drug for luminal A, is on the candidate drug list of C3. The history of TNF-α is closely related to the history of tumor immunotherapy [62]. TNF-α is one of the most important pro-inflammatory cytokines found in breast cancer, mainly secreted by M1 activated macrophages [63]. TNF-α belongs to the TNF/TNFR superfamily and is considered to be one of the most promising anti-cancer factors [64]. TNF-α affects the development of BC at all stages, including the development of primary tumors, EMT, metastasis and disease recurrence [65]. Therefore, a treatment plan related to TNF-α in BC has been produced: neutralization of endogenous TNF-α by TNF antagonists [65]. In some preclinical studies, TNF-α antagonists have been shown to effectively inhibit the growth and spread of BC [65]. The predicted TNF-targeted drugs are MSX-122, nimesulide, adalimumab, AME-527, certolizumab pegol, etanercept, golimumab, infliximab, and thalidomide. Etanercept can bind to TNF-α, thereby inhibiting its biological activity [65]. Infliximab inhibits tumor growth and lymph node metastasis by combining molecular pathways mediated by TNF-α [66]. In addition, when TNF-α stimulates BC, it will activate the transcription factors NFkβ and cJun, drive the transcription of EMMPRIN and MIF genes, and induce the increase of MMP secreted by macrophages in TME. It has been found to promote tumor cell invasion and metastasis [67]. The drugs that target MMP family genes are marimastat, SC-74020, and UK-356618. Among which, marimastat can prevent tumor cell invasion and metastasis [68, 69].

**Table 1:** Information of candidate drugs for each lncRNA subtype

LncRNA subtypes	Numbers of drugs	Numbers of drugs only for one subtype	Numbers of drugs shared with other subtypes	lncRNA subtypes sharing drugs	Drugs shared with other subtypes
C1	33	31	2	C1; C2	BMS-536924
				C1; C2; C6; C7	TW-37
C2	18	13	5	C1; C2	BMS-536924
				C2; C6	AMD-070
					MSX-122
					Plerixafor
				C1; C2; C6; C7	TW-37
C3	45	44	1	C3; C6; C7	R547
C4	39	38	1	C4; C5	Curcumin
C5	16	13	3	C4; C5	Curcumin
				C5; C6	Canfosfamide
					Ezatiostat
C6	34	22	12	C1; C2; C6; C7	TW-37
				C2; C6	AMD-070
					MSX-122
					Plerixafor
				C3; C6; C7	R547
				C5; C6	Canfosfamide
					Ezatiostat
				C6; C7	ABT-737
					Obatoclax
					Phosphonothreonine
					SNS-032
					THZ1
C7	42	35	7	C1; C2; C6; C7	TW-37
				C3; C6; C7	R547
				C6; C7	ABT-737
					Obatoclax
					Phosphonothreonine
					SNS-032
					THZ1

We hypothesized that the presence of multiple single-cell subtypes in a patient is the cause of treatment failure or even tumor recurrence. For patients with multiple subtypes, clinically, the patient may only display the phenotypic characteristics of the dominant subtype, and the phenotypic characteristics of minor/secondary subtypes will be covered up. Only treating the dominant subtype may result in poor treatment effects. For example, lncRNA subtype C1 accounted for the majority/dominance in patient BC05, followed by C7. Clinically, the tumor phenotype characteristics of C1 may cover those of the C7 subtype, and the patient phenotype apparently represents the C1 subtype. Usually, according to the current treatment strategy, BC05 is treated according to the C1 subtype. During the process of treatment, C1 cells are gradually eliminated, and C7 cancer cells appear, leading to drug resistance or a potential risk of recurrence. Therefore, for patients with multiple subtypes, we proposed new treatment strategies that should use targeting multi-subtype drugs or combinations of targeting single-subtype drugs to simultaneously treat the patient with multiple subtypes. For BC05, we can use a drug shared by two subtypes, such as TW-37, which is confirmed to effectively induces apoptosis in a dose-dependent manner [70] or a combination of drugs to target C1 and C7 subtypes, such as pertuzumab [71] and melatonin [72]. Treatment strategies of other patients are in Additional file 7.

## Discussion

Accurate classification can improve the accuracy and success rate of subtype diagnosis, obtain the good prognosis of each subtype, and greatly help the exploration of precise treatment strategies. In our research, we used lncRNA expression profiles of single cells to retype BC to better highlight the heterogeneity among cancer cells, identify precise subtypes, and performing more precise treatment.

PAM50 molecular subtypes are based on bulk sequencing and cannot completely reveal intratumoral heterogeneity. The subtyping of BC at the single-cell level is mainly based on mRNAs/genes, still leaving some room for improvement. In this article, we first demonstrated that lncRNAs are more suitable as markers for presenting the intratumoral heterogeneity of BC by comparing the expression consistency and variance of mRNAs and lncRNAs among cells and found that the expression variance of lncRNAs was higher than that of mRNAs. Therefore, we used single-cell lncRNA expression data to retype 317 tumor cells from 12 samples of 10 BC patients, identified new lncRNA subtypes, their markers and specific functional characteristics, and performed survival analysis. At the same time, we compared lncRNA subtypes with the PAM50 subtype, which is the gold standard for BC classification, and found that lncRNA subtypes can more accurately reveal intratumoral heterogeneity.

When subtyping by PAM50 is to cluster patients, each patient shows one subtype. While subtyping based on single-cell lncRNA markers is the clustering of cells, we also found that some patients had multiple lncRNA subtypes. We also found that some patients had multiple lncRNA subtypes. If these patients with multiple subtypes are treated only for the tumor cells of the major/dominant subtype, neglecting those of minor subtypes based on the current treatment strategy, the activities of these tumor cells of minor subtypes would be highlighted with the disappearance of tumor cells of major subtype in the progress of treatment, which would result in drug resistance and tumor recurrence. Based on this, we further identified candidate therapeutic drug targets and corresponding drugs for each lncRNA subtype to explore new treatment strategies. The literature verification rate of predicted drugs is 86.2%, which reflects the accuracy of our results to some extent. For these patients, we propose a new treatment strategy in which we should use one targeting multi-subtype drug or choose a combination of drugs targeting only one subtype to improve the treatment effect. Specifically, we prefer to recommend the former with fewer side effects. For BC05, we recommend TW-37 shared by C1 and C7 subtypes rather than the combination of pertuzumab and melatonin.

In this article, some positive results have been obtained and also providing new perspectives and foundations for further BC research, but these results need to be verified further, especially by population investigations in the future.

## Supplementary Information

**Additional file 1.** Density matrix.zip.

**Additional file 2.** Results of SC3.xls.

**Additional file 3.** lncRNA markers of lncRNA subtypes.xls.

**Additional file 4.** DEGs of lncRNA subtypes.xls.

**Additional file 5.** GO terms of lncRNA subtypes.xls.

**Additional file 6.** Candidate drugs of lncRNA subtypes.xls.

**Additional file 7.** Treatment strategies of patients.docx.

**Additional file 8: Figure S1.** Expression density maps of PAM50 subtypes in 10 patients. The abscissa represents the level of gene expression, and the ordinate represents the density of the expressed cells. Different colors of the curve represent different PAM50 subtypes of BC.

**Additional file 9: Figure S2.** Heatmap of the expression activity of PAM50 in 10 patients. In almost all samples, multiple subtypes are highly expressed in different cells at single-cell resolution.

**Additional file 10: Figure S3.** Cluster evaluation of SC3 to determine the best cluster number. a). Consensus matrix diagram of the SC3 results. A similarity of 0 (blue) indicates that two cells are always assigned to different clusters. In contrast, a similarity of 1 (red) indicates that two cells are always assigned to the same cluster. b). Average silhouette width of SC3 results. The average silhouette width varies from 0 to 1, where 1 represents a perfectly block-diagonal consensus matrix. The best clustering is achieved when the average silhouette width is close to 1.

## Data Availability

The datasets used in this article is download from Gene Expression Omnibus (GEO) (https://www.ncbi.nlm.nih.gov/geo/query/acc.cgi?acc=GSE75688) with accession number GSE75688 [26]. And all data generated during this study are included in supplementary information files.
