# Supplementary material for: Precision treatment exploration of breast cancer based on heterogeneity analysis of lncRNAs at the single-cell level
Source: BMC Cancer. 2021 Aug 13;21:918. doi: 10.1186/s12885-021-08617-7 (PMC8361656; doi:10.1186/s12885-021-08617-7)
Supplement: Supplementary file 7 — Additional file 7. Treatment strategies of patients.docx. [file 12885_2021_8617_MOESM7_ESM.docx]

**Patient BC01** contains only one single-cell lncRNA subtype C7, and any candidate therapeutic drug of C7 subtype can be used for treatment. A total of 42 drugs, including 7 multi-target drugs (**TW-37** also targets C1, C2 and C6 subtypes; **R547** also targets C3 andC6 subtypes; **ABT-737**, **obatoclax**, **phosphonothreonine**, **SNS-032** and **THZ1** also target C6 subtype) and 35 single-target drugs such as **3'-Oxo-Adenosine**, **4-**Melatonin and so on.

The main single-cell lncRNA subtype of **patient BC02** is C3, and the secondary subtypes are C5 and C6. No drug can target C3, C5, and C6 subtypes at the same time, so drug combinations that target the three subtypes are chosen. There are 3 kinds of combination medication plans:

multi-target drug + multi-target drug (such as **R547+canfosfamide**);

multi-target drug + single-target drug (such as **ezatiostat+APR-246**);

single-target drug + single-target drug (such as **APR-246+carfentanil+AT13387**)

Taking into account drug interactions and drug side effects, we recommend a plan with a smaller amount of drugs.

**Patient BC03** has two single cell subtypes (C2 and C6), of which C6 accounts for the main proportion. There are two treatment options:

1. Use drugs that target both C2 and C6, such as **TW-37**;

2. Combination of drugs that target C2 and C6 subtypes, respectively, such as **A-674563+ EPZ005687**.

Taking into account drug interactions and drug side effects, we recommend first treatment option.

**Patient BC04** contains major/dominant subtype C4 and secondary subtype C2, there are no drugs that target C2 and C4, so we can only use a combination of drugs, such as **A-674563+adalimumab**.

**Patient BC05** contains two subtypes C1 and C5, we should use a drug shared by two subtypes, such as **TW-37**, or a combination of drugs to target C1 and C7 subtypes, respectively, such as **pertuzumab+melatonin**.

**Patients BC06, BC10, and BC11** only contain C5 subtype, any one of the 17 therapeutic drugs of C5 subtype can be chosen, such as multi-target drug (canfosfamide,

ezatiostat and Curcumin) or single-target drug (such as carfentanil, Dezocine and so on).

**Patients** **BC07 and BC08** contain two subtypes, C2 and C5. No drug can target both C2 and C5 subtypes at the same time. A combination of drugs that target C2 and C5 subtypes, respectively, can be chosen, such as **A-674563+asimadoline.**

Supplementary table 7. Treatment strategies for 10 patients

| Patients | lncRNA subtypes | Type of treatment strategies | Treatment plans | examples |
| --- | --- | --- | --- | --- |
| BC01 | C7 | individual drug | multi-target drug | THZ1 |
|  |  |  | single-target durg | Melatonin |
| BC02 | C3, C5, C6 | combination medication | multi-target drug + multi-target drug | R547+canfosfamide |
|  |  |  | multi-target drug + single-target drug | Ezatiostat+APR-246 |
|  |  |  | single-target drug + single-target drug | APR-246+Carfentanil  +AT13387 |
| BC03 | C2, C6 | individual drug | multi-target drug | TW-37 |
|  |  | combination medication | multi-target drug + multi-target drug | BMS-536924+ABT-737 |
|  |  |  | multi-target drug + single-target drug | BMS-536924+AT13387 |
|  |  |  | single-target drug + single-target drug | A-674563+ EPZ005687 |
| BC04 | C4, C2 | combination medication | multi-target drug + multi-target drug | AMD-070+Curcumin |
|  |  |  | multi-target drug + single-target drug | AMD-070+adalimumab |
|  |  |  | single-target drug + single-target drug | A-674563+adalimumab |
| BC05 | C1, C7 | individual durg | multi-target drug | TW-37 |
|  |  | combination medication | multi-target drug + multi-target drug | BMS-536924+THZ1 |
|  |  |  | multi-target drug + single-target drug | BMS-536924+ melatonin |
|  |  |  | single-target drug + single-target drug | pertuzumab+melatonin |
| BC06  BC10  BC11 | C5 | individual drug | multi-target drug | canfosfamide |
|  |  |  | single-target durg | miglustat |
| BC07  BC08 | C2, C5 | combination medication | multi-target drug + multi-target drug | BMS-536924+curcumin |
|  |  |  | multi-target drug + single-target drug | BMS-536924+asimadoline |
|  |  |  | single-target drug + single-target drug | A-674563+asimadoline |
