# Supplementary figures and images for: Precision treatment exploration of breast cancer based on heterogeneity analysis of lncRNAs at the single-cell level
Source: BMC Cancer. 2021 Aug 13;21:918. doi: 10.1186/s12885-021-08617-7 (PMC8361656; doi:10.1186/s12885-021-08617-7)

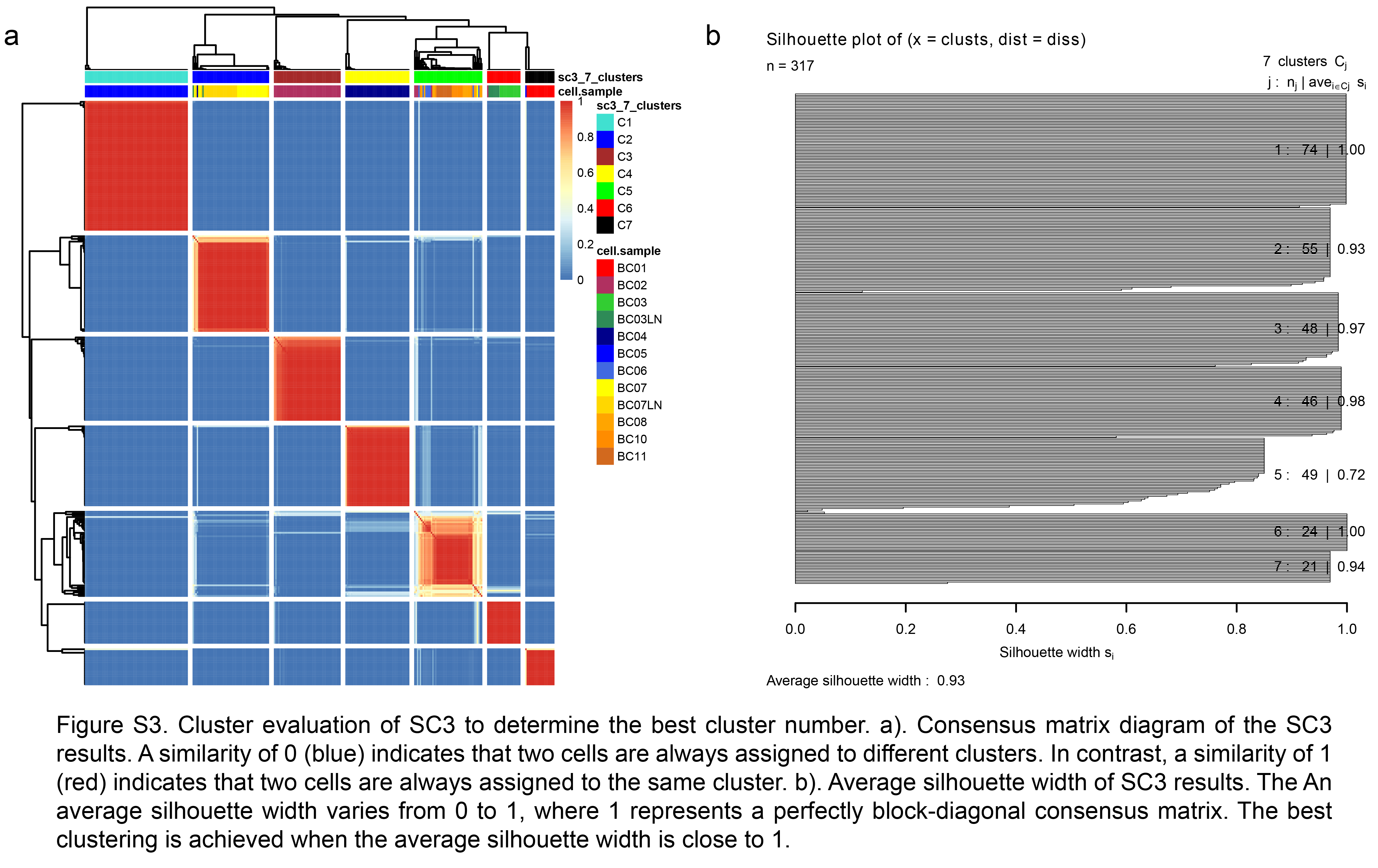

Supplement: Supplementary file 10 — Additional file 10: Figure S3. Cluster evaluation of SC3 to determine the best cluster number. a). Consensus matrix diagram of the SC3 results. A similarity of 0 (blue) indicates that two cells are always assigned to different clusters. In contrast, a similarity of 1 (red) indicates that two cells are always assigned to the same cluster. b). Average silhouette width of SC3 results. The average silhouette width varies from 0 to 1, where 1 represents a perfectly block-diagonal consensus matrix. The best clustering is achieved when the average silhouette width is close to 1. [file 12885_2021_8617_MOESM10_ESM.png]
